# Determining Antibody-Binding Site of Streptococcal Pyrogenic Exotoxin B to Protect Mice from Group A Streptococcus Infection

**DOI:** 10.1371/journal.pone.0055028

**Published:** 2013-01-31

**Authors:** Nina Tsao, Miao-Hui Cheng, Hsiu-Chen Yang, Yu-Chieh Wang, Yi-Ling Liu, Chih-Feng Kuo

**Affiliations:** 1 Department of Biological Science and Technology, I-Shou University, Kaohsiung City, Taiwan; 2 Department of Nursing, I-Shou University, Kaohsiung City, Taiwan; Universidad Nacional de La Plata., Argentina

## Abstract

Streptococcal pyrogenic exotoxin B (SPE B), a cysteine protease, is an important virulence factor in group A streptococcal (GAS) infection. SPE B binds and cleaves antibody isotypes and further impairs the immune system by inhibiting complement activation. In this study, we examined the antibody-binding site of SPE B and used it to block SPE B actions during GAS infection. We constructed different segments of the *spe B* gene and induced them to express different recombinant fragments of SPE B. Using an enzyme-linked immunosorbent assay (ELISA), we found that residues 345–398 of the C-terminal domain of SPE B (rSPE B_345–398_), but not the N-terminal domain, was the major binding site for antibody isotypes. Using a competitive ELISA, we also found that rSPE B_345–398_ bound to the Fc portion of IgG. The *in vitro* functional assays indicate that rSPE B_345–398_ not only interfered with cleavage of antibody isotypes but also interfered with SPE B-induced inhibition of complement activation. Immunization of BALB/c mice using rSPE B_345–398_ was able to induce production of a high titer of anti-rSPE B_345–398_ antibodies and efficiently protected mice from GAS-induced death. These findings suggest that SPE B uses its C-terminal domain to bind the Fc portion of IgG and that immunization of mice with this binding domain (rSPE B_345–398_) could protect mice from GAS infection.

## Introduction

*Streptococcus pyogenes* (group A streptococcus; GAS) is an important human pathogen that causes a variety of diseases, including pharyngitis, cellulitis, impetigo, scarlet fever, necrotizing fasciitis, puerperal sepsis, and streptococcal toxic shock syndrome (STSS) [1], [2], [3]. Despite intensive care with antimicrobial therapy, the mortality rate has remained high, as has the incidence post-infection sequelae, such as acute rheumatic fever [4]. Several virulence factors have been reported that contribute to evasion of host immunity by GAS. These factors consist of the cell surface M protein, M-like protein, the hyaluronic acid capsule, the streptococcal inhibitor of complement, and C5a peptidase [5],[6],[7],[8],[9], as well as secreted exotoxins and enzymes such as streptococcal pyrogenic exotoxin B (SPE B), IdeS (IgG-degrading enzyme of *S*. *pyogenes*), endo-β-N-acetylglucosaminidase (EndoS), and DNases [10], [11], [12], [13], [14].

SPE B, a cysteine protease secreted by most strains of GAS, has been reported to play a role in the pathogenesis of various diseases [10], [15], [16], [17]. SPE B is expressed as a 40 kDa precursor zymogen (zSPE B) and is subsequently cleaved to a 28 kDa mature form protease (mSPE B) [10]. mSPE B directly interferes with host immunity by degrading complement molecules [18], [19] and antibodies [10], [20], [21] and also digests other host proteins, such as fibronectin, vitronectin, kininogen, metalloprotease, prointerleukin-1β, and the urokinase receptor [22], [23], [24], [25], [26]. Another report has suggested that the *covS* mutation switches the M1T1 strain GAS phenotype from speB^high^/speA^−/^Sda1^low^ to the highly virulent speB^−/^speA^+^/Sda1^high^ phenotype [27]. Several reports still indicate that a *speB* mutant strain decreases resistance to neutrophil phagocytosis, dissemination to organs, and mortality in a mouse model [16], [21], [28]. Our previous study also indicates that SPE B and streptolysin (SLS) have a synergistic effect on GAS-mediated macrophage death and the resistance of GAS to immune cell-mediated killing and that SPE B plays a more important role than SLS in increasing the severity of GAS-induced skin lesions [29]. Clinical investigation indicates that high levels of SPE B protease activity are significantly associated with signs of STSS and with mortality. Patients with lower antibody levels against SPE B are more likely to succumb to invasive GAS disease [30]. Taken together, these reports indicate that SPE B is a critical virulence factor in GAS infection.

SPE B has been known to digest free immunoglobulins, including IgG, IgA, IgM, IgE, and IgD [12], as well as antigen-bound IgG [20], [21]; hence, antibody-mediated neutralization and complement activation in GAS infection are impaired by SPE B. However, the exact antibody-binding site of SPE B has yet to be clearly defined. In this study, we demonstrated that SPE B uses its C-terminal domain, specifically amino-acid residues 345–398, to bind the Fc portion of IgG. Using a recombinant rSPE B_345–398_ protein to block the binding between SPE B and antibody isotypes inhibited cleavage of antibodies by SPE B and SPE B-mediated inhibition of complement activation. Recombinant rSPE B_345–398_ could potentially serve as a vaccine to protect mice from GAS-induced death.

## Materials and Methods

### Purification of Human Immunoglobulins

Normal human sera were donated by healthy volunteers. We obtained written informed consent from each person and approved by the ethics committee of E-Da Hospital. Protein L-agarose (Thermo) and protein A-agarose (Thermo) were used to purify human serum immunoglobulins. Ten milliliters of binding buffer containing 0.1 M phosphate and 0.15 M sodium chloride (pH 7.2) was added to a protein L- agarose-packaged column. Normal human sera diluted 2-fold with binding buffer were passed through the protein L column. IgG, IgM, IgA, IgE, and IgD bound to protein L- agarose because of the ability of protein L to bind the κ chain of immunoglobulins. After washing with binding buffer to remove unbound materials, 6 to 10 ml of the elution buffer containing 0.1 M glycine (pH 2.5) was added to elute the five immunoglobulin isotypes. The immunoglobulin mixture was then dialyzed using vivaspin 20 (GE Healthcare) with the binding buffer for protein A-agarose that contained 20% phosphate-buffered saline (PBS). The immunoglobulin mixture was passed through the protein A column to purify IgG, which was purified prior to use. Unbound immunoglobulin isotypes were also collected, and the contents were further examined by western blotting using anti-isotype antibodies. In this unbound mixture, only IgM and IgA were detected. The concentrations of IgE and IgD were below the level of detection for the assay. We prepared the IgM-IgA mixture for further use.

### Cloning and Expression of SPE B Truncations

The recombinant SPE B and the C192S mutant lacking protease activity were prepared as described previously [19], [31]. Briefly, the genomic DNA of GAS was extracted and the structural gene of SPE B was amplified using polymerase chain reaction (PCR) with the sense primer 5′-GGATCCGGATCCCATCATCATCATCATCATGATCAAAACTTTGCTCGTAACGAA-3′ and antisense primer 5′-GGATCCGGATCCCTAAGGTTTGATGCCTACAACAG-3′. The PCR product was purified and then cloned into the *Bam*H1 site of pET-21a vector. The wild-type construct was further used to produce C192S mutation using overlap extension PCR. Four pairs of specific primers with *Bam*H1 and *Xho*1 restriction sites were designed to make the SPE B truncated proteins; for *speB*_146–280_, 5′-CGCGGATCCCAACCAGTTGTTAAATCT-3′ and 5′-CCGCTCGAGACTAGATGGACCATAATCCAT-3′; for *speB*_146–398_, 5′-CGCGGATCCCAACCAGTTGTTAAATCT-3′ and 5′-CCGCTCGAGAGGTTTGATGCCTACAAC-3′; for *speB*_281–358_, 5′-GCGGATCCGGTTCTGCAGGTAGCTCTC-3′ and 5′-CCGCTCGAGACCCCAGTTAACATGGTAGA-3′; for *speB*_345–398_, 5′-GCGGATCCGATGGTGCTGACGGACG-3′ and 5′-CCGCTCGAGAGGTTTGATGCCTACAACA-3′. The PCR products were purified and then cloned into the *Bam*H1 and *Xho*1 restriction sites of the pET-42a vector. The recombinant plasmids were transformed into *Escherichia coli* BL21(DE3) pLyS strains. Recombinant proteins that contained N-terminal glutathione S-transferase (GST) and six histidines at the C-terminal end were produced by growing cells at 37°C overnight in LB medium containing 100 mg of isopropyl-β-D-thiogalactoside, 25 mg of kanamycin, 16 g of Bacto-Peptone, 8 g of Bacto-yeast extract, and 16 g of NaCl in 1 liter. Proteins were then purified using Ni^2+^-chelating chromatography (GE Healthcare). The purified recombinant proteins were further examined by 12% sodium dodecyl sulfate-polyacrylamide gel electrophoresis (SDS-PAGE) and confirmed by western blotting using rabbit anti-SPE B serum (kind gift from Dr. Y. S. Lin, National Cheng Kung University Medical College, Taiwan.), and 2 µg of bovine serum albumin (BSA) (Sigma) was used as a control.

### Examining Binding of SPE B to Immunoglobulins

One gram of the CNBr-activated sepharose 4B gel (Pharmacia Biotech) was suspended in 250 ml of 1 mM HCl for 30 min at 4°C. The gel was then washed with alternating 750 ml of 1 mM HCl, 100 ml of distilled water, and 300 ml of the reaction buffer containing 0.1 M NaHCO_3_ and 0.5 M NaCl (pH 8.0). The gel was suspended in 15 ml of the reaction buffer (pH 8.0) and was incubated with 10 mg of C192S, the mutant SPE B that lacks protease activity, at 4°C overnight. The coupled gel was washed with 250 ml of 1 M glycine/reaction buffer (pH 8.0) to block unused activated sites. Thereafter, the coupled gel was washed three times with alternating 200 ml of borate buffer containing 0.1 M boric acid and 1 M NaCl (pH 8.5), 100 ml of distilled water, 200 ml of acetate buffer containing 0.1M sodium acetate and 1 M NaCl (pH 4.0), and 100 ml of distilled water. The gel was then suspended in PBS [19].

One milliliter of normal human serum was passed through a CNBr-activated sepharose 4B-packed affinity column that was immobilized with C192S. Buffer containing 50 mM Tris (pH 7.0) was used to wash non-binding materials. After several washes, binding ligands of C192S were eluted with 0.1 M glycine (pH 3.0), and different fractions were collected from the point when the signal appeared on the recording graph by measuring the absorbance at 280 nm [18], [19]. The contents of fraction 13 (F13) and purified immunoglobulins were verified using 12% SDS-PAGE and then transferred to a PVDF membrane (Millipore). After blocking, blots were developed with goat anti-human IgG (Calbiochem), IgM (Abcam), or IgA (Abcam) antibody that was diluted 5000-fold with PBS. Blots were then hybridized using horseradish peroxidase-conjugated rabbit anti-goat IgG (Calbiochem), and the protein bands were visualized using enhanced chemiluminescence (Amersham Biosciences).

### Detecting Cleavage of Immunoglobulins by SPE B

The purified human IgG or the IgM-IgA mixture (400 µg/ml) was incubated with 20 µg/ml of mSPE B or C192S at 37°C for 1, 2, or 18 h with 5 mM dithiothreitol (DTT)/0.1 mM EDTA that was used to activate SPE B protease activity [18], [19]. In another experiment, the purified human IgG or the IgM-IgA mixture (40 µg/ml) was incubated with 2 µg/ml of mSPE B in the absence or presence of different concentrations of rSPE B_345–398_ (10, 20, or 40 µg/ml) at 37°C for 1 h with 5 mM dithiothreitol (DTT)/0.1 mM EDTA. The reaction mixtures mentioned above were separated using 12% SDS-PAGE and then detected by western blot with goat anti-human IgG (Calbiochem), IgM (Abcam), or IgA antibody (Abcam).

### Enzyme-linked Immunosorbent Assay (ELISA)-type Binding Assay

The microtiter plate wells (NUNC) were coated with 2 µM of C192S or BSA (Sigma) in 50 µl of coating buffer containing 100 mM of NaHCO_3_ and 100 mM of Na_2_CO_3_ (pH 9.0) at 37°C for 1 h, and blocked with 200 µl of 5% skim milk (Sigma) at 4°C overnight. After several washes with 0.1% Tween-20/PBS (PBS-T), 50 µl of human serum diluted 100-fold with PBS was added into the wells and incubated at 4°C overnight. After several washes, 50 µl of peroxidase-conjugated goat anti-human IgG, IgM, or IgA antibody (Millipore) (1∶10000) was added and incubated at 37°C for 1 h. Next, the TMB substrate (Vector Laboratories) was added, and the absorbance values were read at 650 nm. In a direct binding assay for Fc fragment, 50 µl of different concentrations of human Fc fragments of human IgG (Millipore) (0.5–5 µM) or purified human IgG (1 µM) were added to the C192S- or rSPE B_345–398_- coated wells. After that, peroxidase-conjugated goat anti-human IgG antibody (Millipore) (1∶10000) was added and then the TMB substrate (Vector Laboratories) was developed, and the absorbance values were read at 650 nm.

Alternatively, for detection of immunoglobulins binding with SPE B recombinant proteins, the microtiter plate wells were coated with either 0.1 µg of purified human IgG or the IgM-IgA mixture in 50 µl of coating buffer (pH 9.0) at 37°C for 1 h. The plates were washed and then blocked with 200 µl of 1% BSA/PBS at 4°C overnight. After washing, 50 µl of the various SPE B recombinant proteins (2 µM) were added into the plates and incubated at 4°C overnight. After several washes with 0.1% PBS-T, mouse anti-GST monoclonal antibody (Abcam) (1∶5000) was added and incubated for 1 h followed incubation by 100 µl of peroxidase-conjugated goat anti-mouse IgG antibodies (Calbiochem) (1∶10000). After several wash with 0.1% PBS-T, the TMB substrate was added, and the absorbance values were read at 650 nm. The results of three experiments are represented and expressed as the mean ± standard deviation.

### Inhibition ELISA

The microtiter plate wells (NUNC) were coated with 2 µM of purified rSPE B_345–398_ or BSA in 50 µl of the coating buffer containing 100 mM NaHCO_3_ and 100 mM Na_2_CO_3_ (pH9.0) at 37°C for 1 h. The plates were washed and then blocked with 200 µl of 1% BSA/PBS at 4°C overnight. Different concentrations of protein A or protein L (0.1–1 µg/ml) were add with purified human IgG (1 µg/ml) at varying molar ratio (0.5–5) and incubated at 37°C for 30 min, and was then added to rSPE B_345–398_ or BSA-coated microtiter plates and incubated at 4°C overnight. After washing with 0.1% PBS-T, peroxidase-conjugated goat anti-human IgG antibody (Millipore) (1∶10000) was added. Next, the TMB substrate (Vector Laboratories) was added, and the absorbance values were read at 650 nm. Relative binding activity was calculated as follows: activity = 100% × (*A _650_* (sample)-*A _650_*(BSA control))/(*A _650_* (IgG only sample)-*A _650_* (BSA control)).The results of three experiments are represented and expressed as the mean ± standard deviation.

### Assessment of Complement Functional Activity

Complement activation through the classical pathway by human serum was detected by the Wielisa COMPL300 Total Complement Functional Screen kit (Wieslab AB) [19]. The kit provides strips of wells precoated with IgM for evaluation of the classical pathway. Following the kit instructions, the serum of a healthy individual, provided by the kit, was diluted 100-fold in specific buffers [19]. The diluted serum was then incubated with 20 µg/ml of mSPE B in the absence or presence of different concentrations (5, 10, or 20 µg/ml) of rSPE B_345–398_ for 15 min in a 37°C water bath. Thereafter, mSPE B-treated human serum was added to strips and incubated for 1 h at 37°C. Alkaline phosphatase-conjugated anti-human C5b-9 antibody, provided by the kit, was added and incubated for another 30 min at room temperature. Finally, the substrate was added, and the absorbance values were read at 405 nm. In each assay, standard positive and negative control sera were provided in the kit. Complement activity was calculated as follows: activity = 100% × (*A _405_* (sample)-*A _405_* (negative control))/(*A _405_* (standard serum)-*A _405_* (negative control)) [18], [19]. In each assay, samples, standard serum, and negative control serum were tested in duplicate at a fixed dilution. The percent inhibition was calculated as follows: 100% × [1-(complement activity of sample)/(complement activity of standard serum)]. The results of three experiments are represented and expressed as the mean ± standard deviation.

### rSPE B_345–398_ Protection Assay in GAS-infected Mice

BALB/cByJNarl mice were purchased from the National Laboratory Animal Center in Taiwan. They were maintained on standard laboratory chow and had access to water ad libitum in the animal center at I-Shou University. The animals were raised and cared for in accordance with the guidelines established by the National Science Council of the Republic of China. All procedures and the care and handling of the animals were reviewed and approved by the Institutional Animal Care and Use Committee at I-Shou University. 7- to 8-week old male mice were used in all experiments.

Either PBS or 400 µg/ml of rSPE B_345–398_ was mixed with an equal volume of Freund’s complete adjuvant (Sigma-Aldrich), and then 0.25 ml of the emulsion was inoculated intraperitoneally into BALB/c mice. After that, 25 µg of rSPE B_345–398_ emulsified with Freund’s incomplete adjuvant (Sigma-Aldrich) was administered intraperitoneally every two weeks for a total of three boosts. The sera of mice were collected after immunization, and the titer of anti- rSPE B_345–398_ antibody of each mouse was determined by ELISA, as previously described. After immunization, groups of 8 to 10 BALB/c mice were injected subcutaneously with 1 ml of air for two consecutive days to form an air pouch. Two days later, 0.2 ml of bacterial suspension containing 2.5 × 10^8^ colony forming units (CFU) of *S. pyogenes* NZ131 was inoculated into the air pouch [29], and the animals were observed every day for a total of 14 days. During the observation time, the degree of skin lesion was photographed and measured using ImageJ 1.410 software (National Institutes of Health) [29]. The average damage area in each group was generated by examining the skin lesions from 8 to 10 mice.

### Statistics

Statistical analysis was performed using ANOVA. Differences were considered significant at *P*<0.05. For the mouse model, the survival curves were compared for significance using the log-rank test.

## Results

### C192S-bound Antibodies Present in Human Sera

**Figure 1:**
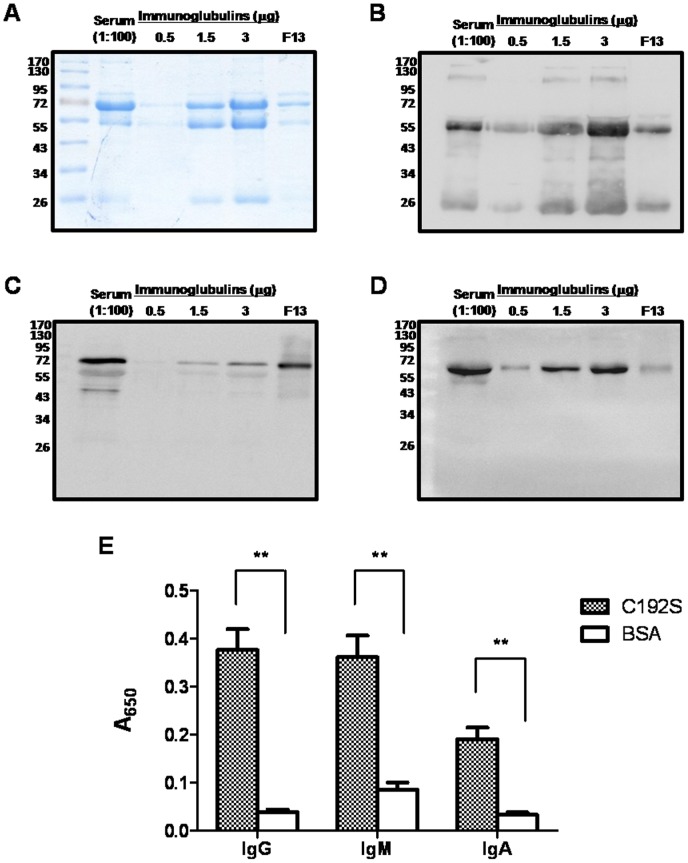
Binding of serum immunoglobulins and C192S. Human sera were passed through an affinity column immobilized with C192S, and then the binding ligands of C192S were eluted and the fraction 13 (F13) was collected. The contents in diluted human serum, different amount of purified human immunoglobulins, and F13 (1 µg) were verified using 12% SDS-PAGE (A) and blotted with goat anti-human IgG (B), IgM (C), or IgA (D). (E) 2 µM of purified C192S or BSA was coated in a 96-well ELISA plate, and binding of IgG, IgM, or IgA with C192S or BSA was detected by ELISA, as described in Materials and Methods. **P<0.01 compared with values determined for the BSA group.

To examine the binding of SPE B with antibodies in human sera, the SPE B mutant protein C192S was used because of the instability of the active form of SPE B (mSPE B) [18], [19]. Human sera were passed through the affinity column packed with C192S immobilized on sepharose 4B. Different fractions were collected during the elution and their contents were analyzed by western blotting using anti-human IgG, IgM or IgA. When compared with protein L-purified human IgG, IgM and IgA, we found that fraction 13 (F13) contained human IgG, IgM, and IgA and that the amount of IgG in the F13 fraction is most abundant, IgM is less, and IgA is least abundant (Figure 1A–D). No IgD or IgE was found in the F13 fraction (data not shown) because of the low concentration of both isotypes in human sera. We observed similar results with an ELISA assay; the serum IgG and IgM binded more strongly to the C192S than IgA did (Figure 1E). These results indicate that C192S was able to efficiently bind IgG, IgM, and IgA in human sera (Figure 1).

### SPE B Cleaved IgG, IgM, or IgA

**Figure 2:**
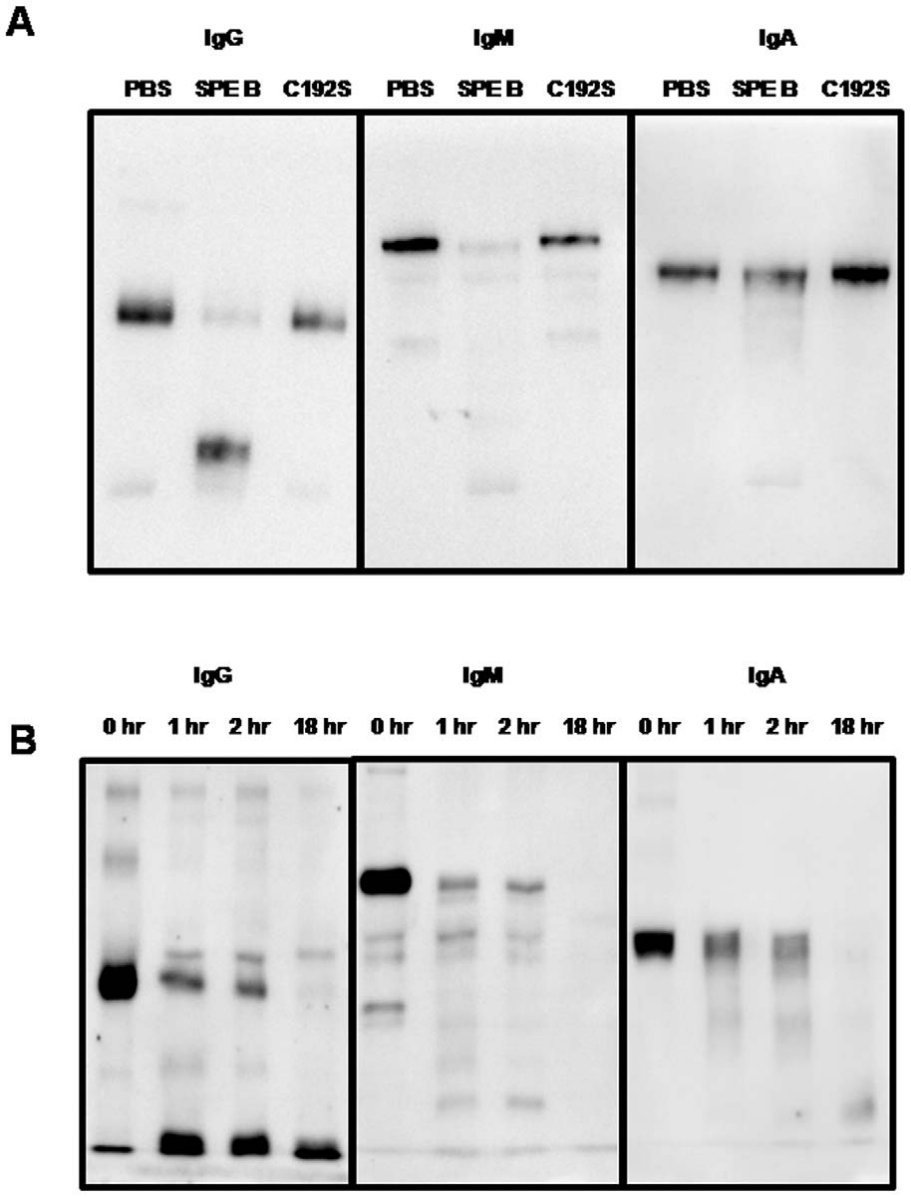
Effect of SPE B on immunoglobulins. (A) Either 400 µg/ml of purified IgG or the IgM-IgA mixture was incubated with 20 µg/ml of mSPE B or C192S for 1 h with 5 mM DTT-0.1 mM EDTA. The reaction mixture was separated using 12% SDS-PAGE and blotted using goat anti-human IgG, IgM, or IgA, as described in Materials and Methods. (B) Either 400 µg/ml of purified IgG or the IgM-IgA mixture was incubated with 20 µg/ml of mSPE B for 1, 2 or 18 h with 5 mM DTT-0.1 mM EDTA. The reaction mixture was separated using 12% SDS-PAGE and blotted using goat anti-human IgG, IgM, or IgA, as described in Materials and Methods.

The purified human IgG, or the IgM-IgA mixture were incubated with the active form mSPE B or C192S mutant at a ratio of 20∶1 (w/w) for different times, and the reaction mixture was separated using SDS-PAGE and blotted with anti-human IgG, IgM, or IgA antibody. After the 1 h incubation, the amount of IgG and IgM were significantly reduced than IgA after mSPE B but not C192S treatment (Figure 2A). The cleavage of antibodies by mSPE B, regardless of the isotype, was almost complete after the 18 h incubation (Figure 2B). These results suggest that active SPE B was able to bind serum IgG, IgM, or IgA and cleave them effectively.

### SPE B Bound Serum Antibodies by its C-terminal Domain

**Figure 3:**
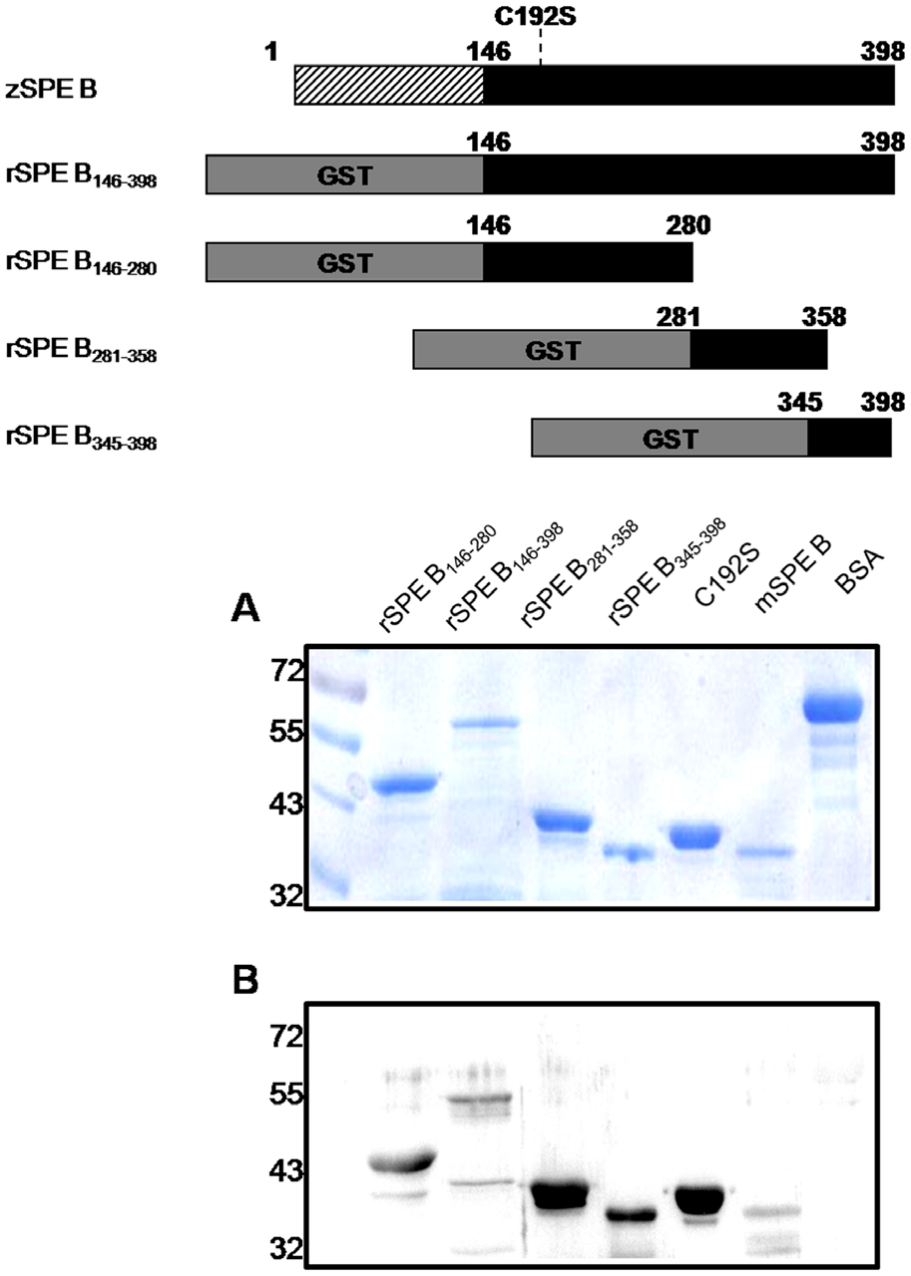
Expression of SPE B truncations. Cloning and expression of different *speB* gene segments were described in Materials and Methods. zSPE B represents zymogen SPE B. (A) Different recombinant SPE B fragments (rSPE B_146–280_, rSPE B_146–398_, rSPE B_281–358_, rSPE B_345–398_), C192S, mSPE B, or BSA were verified by 12% SDS-PAGE, and their molecular weights are shown. (B) The antigenicity of different rSPE B fragments, C192S, mSPE B was determined by western blotting with anti-SPE B antibody, as described in Materials and Methods.

We cloned four plasmids (pSPE B_146–398_, pSPE B_146–280_, pSPE B_281–358_, or pSPE B_345–398_) that contained different *speB* gene segments flanked with GST sequences and six-histidines. pSPE B_146–398_ encoded rSPE B_146–398_ that represented mSPE B; rSPE B_146–280_ lacks the C-terminal domain of mSPE B; both rSPE B_281–358_ and rSPE B_345–398_ lack the N-terminal domain of mSPE B. These purified recombinant proteins were detected with SDS-PAGE. Their molecular weights are shown in Figure 3A, and their antigenicity was confirmed by western blotting with anti-SPE B antibody (Figure 3B).

**Figure 4:**
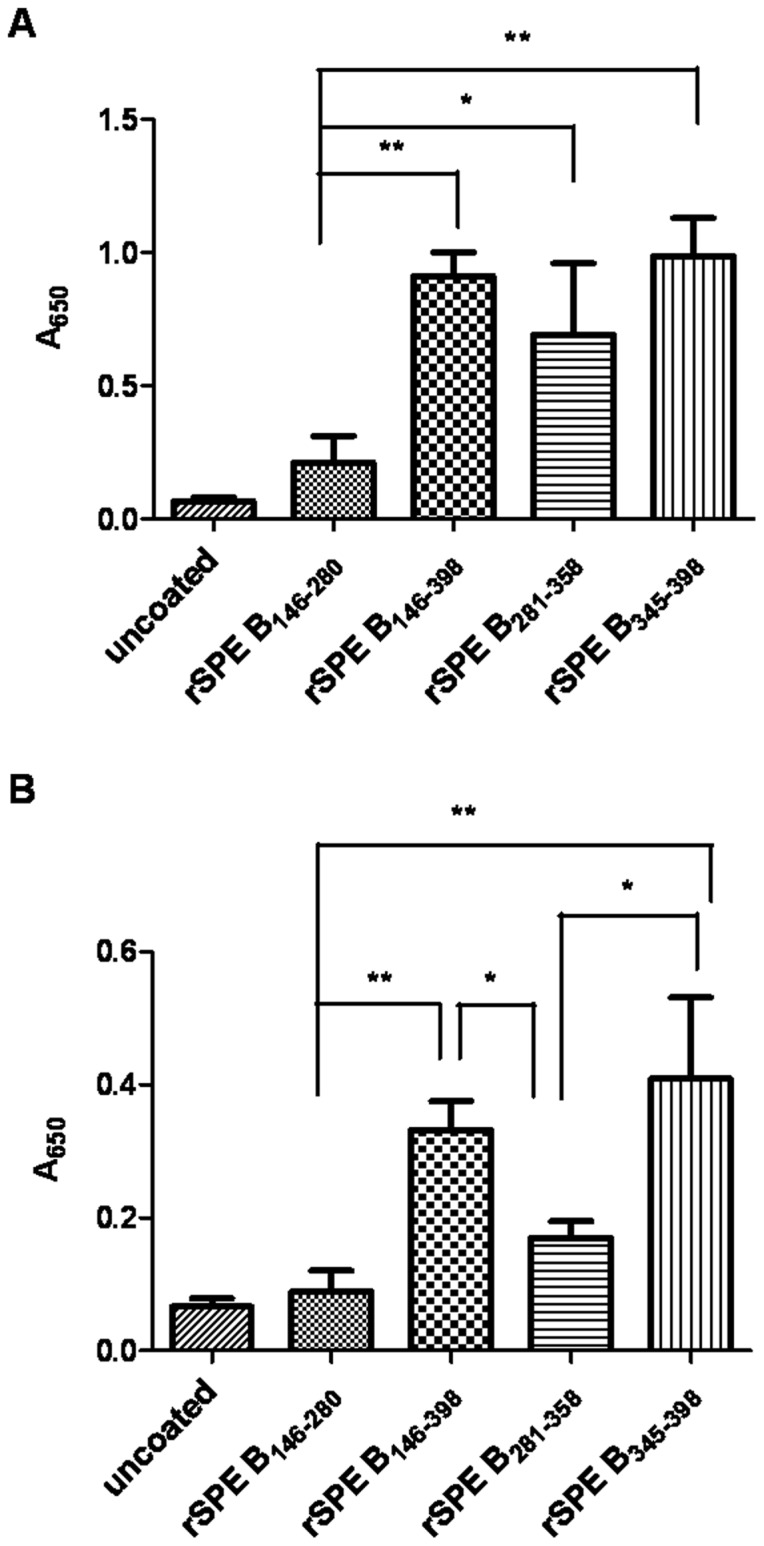
Binding of SPE B truncations and immunoglobulins. Either purified human IgG (A) or the IgM-IgA mixture (B) was coated in a 96-well ELISA plate, and binding of different SPE B truncations with IgG or the IgM-IgA mixture was detected by ELISA, as described in Materials and Methods. *P<0.05, **P<0.01 compared with values determined for the rSPE B_146–280_ or the rSPE B_281–358_ group.

The antibody binding motif of SPE B was further determined with these recombinant SPE B fragments by ELISA assay. The rSPE B_146–398_, representing mSPE B, was able to bind IgG or the IgM-IgA mixture but with different binding capacities (Figure 4). Regardless of IgG or the IgM-IgA mixture, the binding activity of rSPE B_146–280_, which contains the N-terminal domain of mSPE B, had a poor binding capacity to antibody isotypes. Dissecting the C-terminal domain of mSPE B, we found that rSPE B_345–398_ had the strongest binding activity to IgG or the IgM-IgA mixture (Figure 4A and 4B). However, rSPE B_281–358_ manifested stronger binding activity to IgG than N-terminal rSPE B146–280 (Figure 4A).These results suggest that the C-terminal domain, especially amino-acid residues 345–398, of SPE B is the major site for antibody binding.

### The C-terminal Domain of SPE B Bound the Fc Portion of IgG

**Figure 5:**
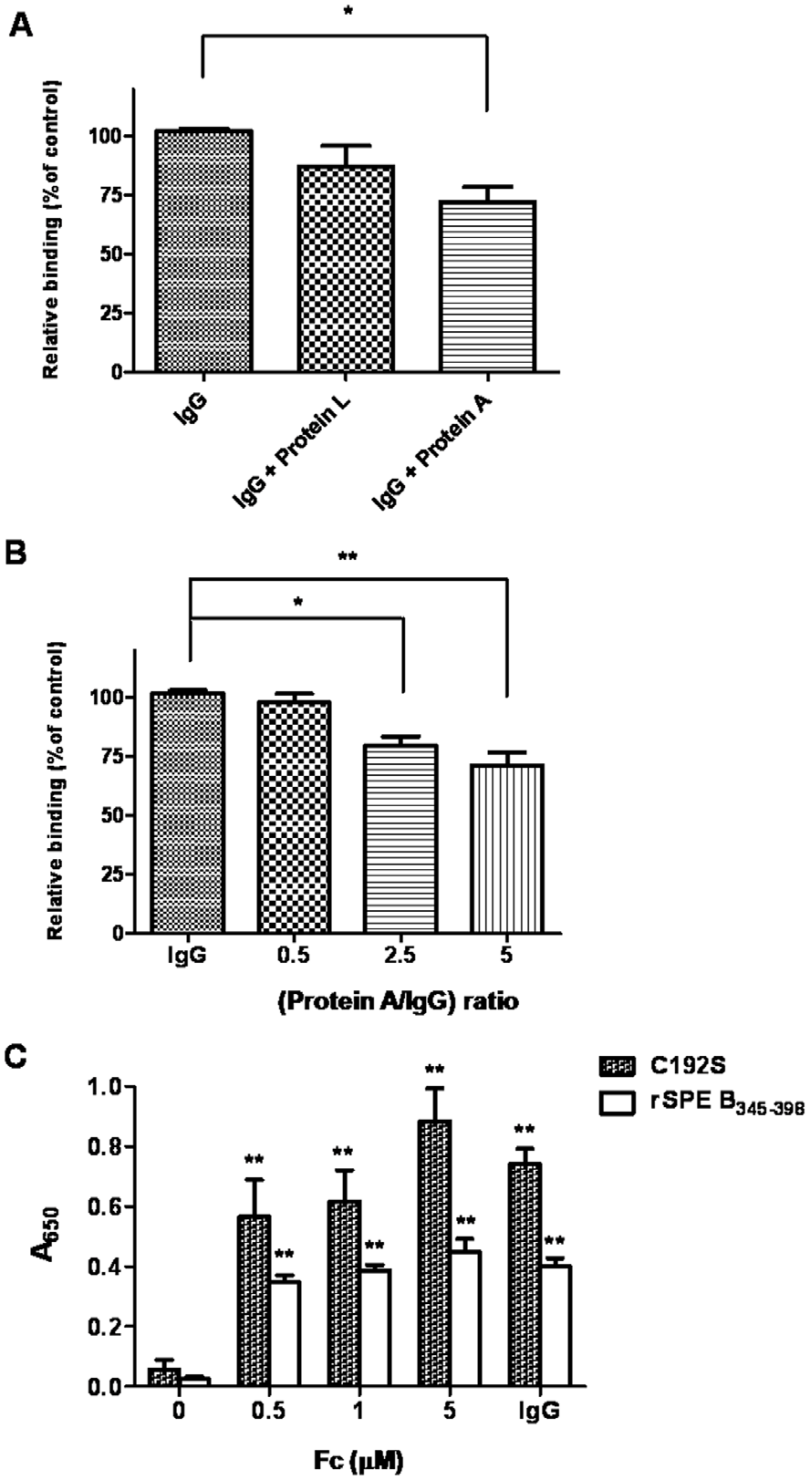
Binding of rSPE B_345–398_ and human IgG. (A) Either protein L or protein A was incubated with purified human IgG at a molar ratio of 2.5 for 30 min at 37°C. (B) Protein A was incubated with purified human IgG at different molar ratios for 30 min at 37°C. The reaction mixtures mentioned above were added to microtiter plates that were pre-coated with purified rSPE B_345–398_ or BSA. Absorbance values were read at 650 nm, and the relative binding activity was calculated as described in Materials and Methods. *P<0.05, **P<0.01 compared with values determined for IgG only group. (C) Purified human IgG or the different concentrations (0.5–5 µM) of Fc fragment of human IgG were added to microtiter plates that were pre-coated with purified rSPE B_345–398_ or C192S, as described in Materials and Methods. Absorbance values were read at 650 nm. **P<0.01 compared with values determined for PBS group.

We chose IgG, the most abundant antibody in human serum, to examine its binding motif with the C-terminal domain of SPE B (rSPE B_345–398_). We used protein L and protein A to compete with the Fab and Fc portions of IgG binding to rSPE B_345–398,_ respectively. The results indicate that protein A but not protein L could inhibit IgG binding to rSPE B_345–398_ (Figure 5A) and that the competitive inhibition was dose-dependent for protein A (Figure 5B). Even at 2 µg per ml, the molar ratio of protein L/IgG was 10, protein L still could not interfere with IgG binding to rSPE B_345–398_ (data not shown). Moreover, a direct ELISA binding assay of purified IgG Fc fragment with rSPE B_345–398_ or C192S also indicate that SPE B uses its C-terminal domain to bind the Fc portion of IgG (Figure 5C).

### rSPE B_345–398_ Interferes with Cleavage of Immunoglobulins and Inhibition of Complement Activation by SPE B

**Figure 6:**
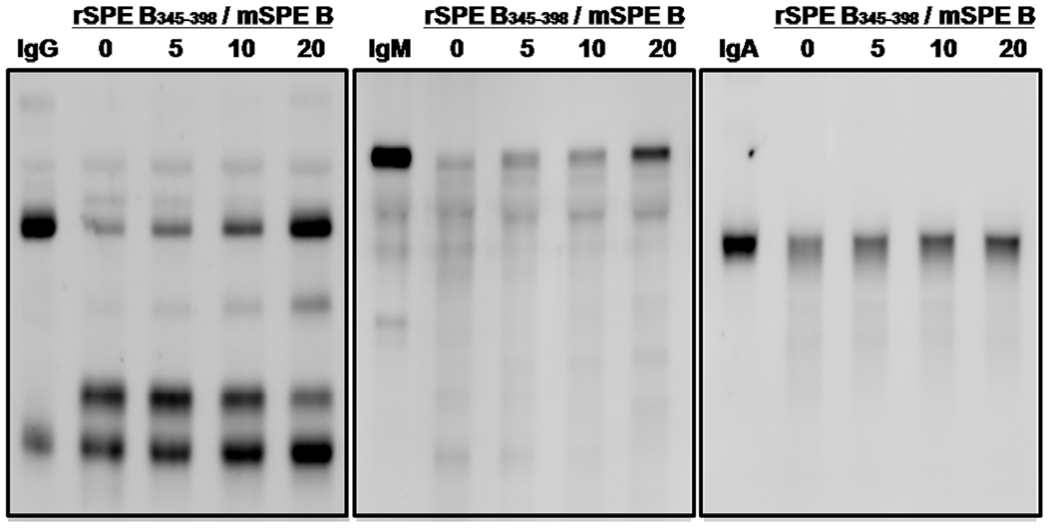
rSPE B_345–398_ interfered with SPE B cleaving immunoglobulins. Either 400 µg/ml of purified human IgG or the IgM-IgA mixture was incubated with 20 µg/ml of mSPE B in the absence or presence of different concentrations of rSPE B_345–398_ at 37°C for 1 h with 5 mM DTT-0.1 mM EDTA. The reaction mixtures were separated using 12% SDS-PAGE and then detected by western blotting with goat anti-human IgG, IgM, or IgA antibodies. Lane 1, purified immunoglobulins; lane 2, mSPE B-treated immunoglobulins; lane 3–5, SPE B-treated immunoglobulins incubated with different concentrations of rSPE B_345–398_; in lane 3, 4, and 5 the molar ratio of rSPE B_345–398_/mSPE B was 5, 10, and 20, respectively.

**Figure 7:**
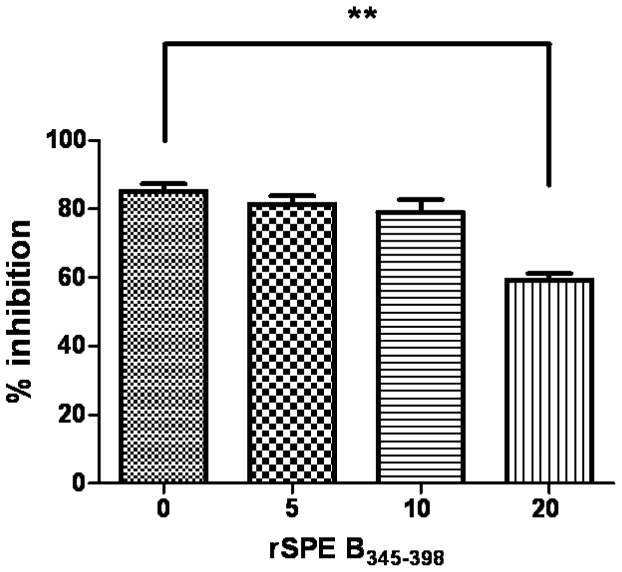
Effect of rSPE B_345–398_ on SPE B-mediated inhibition of classical complement activation. The human sera diluted with specific buffers provided by the Wielisa COMPL300 Total Complement Functional Screen kit were incubated with 20 µg/ml of mSPE B in the absence or presence of different concentrations of rSPE B_345–398_, and the complement activation by human sera was detected using an ELISA assay. The percent inhibition of complement activity was calculated as described in Materials and Methods.

The active form of mSPE B can efficiently cleave IgG, IgM, or IgA within 1 h of incubation (Figure 2A). To confirm that the amino acid sequence 345–398 is the major binding site of mSPE B, rSPE B_345–398_ was used as an inhibitor to interfere with mSPE B binding to antibody isotypes. As shown in Figure 6, rSPE B_345–398_ could effectively inhibit cleavage of antibody isotypes by mSPE B in a dose-dependent manner. Our previous study indicates that mSPE B is able to impair the classical pathway of complement activation [19]. To examine whether rSPE B_345–398_ was able to interfere with mSPE B -mediated inhibition of classical complement activation, a simple ELISA-based assay for the classical pathway was used [19]. The results show that when the dose of rSPE B_345–398_ was increased up to 20 µg/ml, mSPE B-mediated inhibition of classical complement activation dropped from 85% to 59% (Figure 7), indicating that rSPE B_345–398_ could restore complement activation even when mSPE B is present.

### Immunization of rSPE B_345–398_ could Protect Mice from GAS Infection

**Figure 8:**
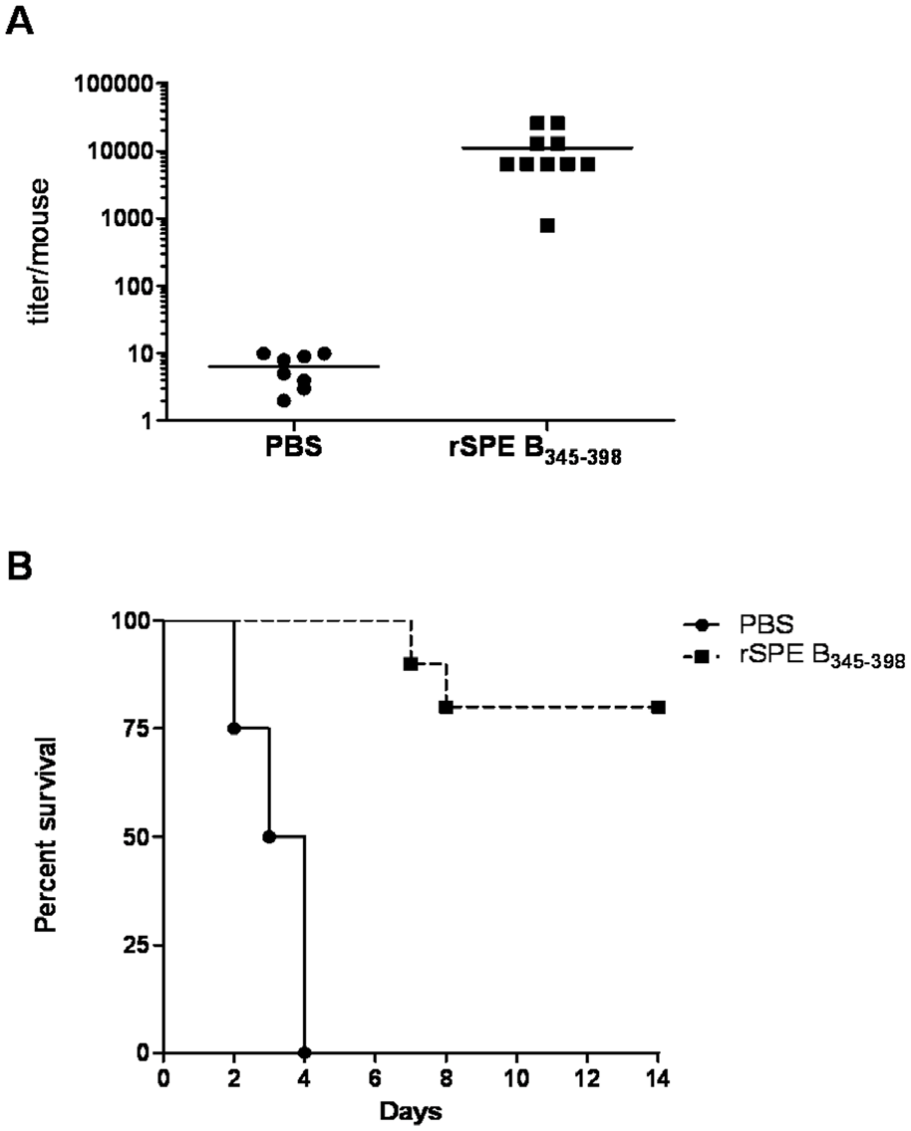
Immunization of rSPE B_345–398_ protects mice from GAS-induced death. (A) The immunization protocol was described in Materials and Methods. Sera were collected, and the titer of anti-rSPE B_345–398_ antibody was detected by ELISA, as described in Materials and Methods. (B) The survival rates in PBS- or rSPE B_345–398_-immunized mice after infection with NZ131 GAS strains. Groups of 8 to 10 BALB/c mice were immunized with PBS (n = 8) or rSPE B_345–398_ (n = 10) and were then inoculated via the air pouch route with 2.5 × 10^8^ CFU of GAS strain NZ131. The survival curves were compared for significance using the log-rank test for the rSPE B_345–398_ versus the PBS group (*P*<0.01).

Immunization of BALB/c mice using rSPE B_345–398_ was able to induce production of a high titer of anti-rSPE B_345–398_ antibodies (Figure 8A). To examine whether anti-rSPE B_345–398_ antibodies possess neutralizing activity to block mSPE B actions, we infected control mice and rSPE B_345–398_-immunized mice with a lethal dose of GAS strain NZ131 (2.5 × 10^8^ CFU/mouse) through the air-pouch route, which mimics local necrotizing fasciitis [29], and monitored survival. The results indicate that control mice all died within 4 days after GAS infection, while 80% of rSPE B_345–398_-immunized mice were still alive 14 days after GAS infection (Figure 8B). Moreover, the skin lesions induced by GAS infection were smaller in rSPE B_345–398_-immunized mice (81±21 mm^2^) compared to control mice (187±38 mm^2^). These results indicate that rSPE B_345–398_ could be used as a vaccine to block mSPE B actions and efficiently protect mice from GAS infection.

## Discussion

Several reports suggest that SPE B contributes to increasing GAS invasion [10], [16], [17], [29], degrading fibronectin, vitronectin, and fibrinogen [22], [32], cleaving urokinase plasminogen activator receptor [26], activating matrix metalloprotease [24], generating active IL-1β from its precursor [25], damaging endothelial cells [16], and reducing the phagocytic activity of U937 monocytic cells [33]. Our previous studies using an air pouch animal model show the correlation of SPE B with GAS-induced tissue damage, bacterial dissemination and bacteremia [16], [29]. In addition, SPE B has been known to directly interfere with killing by the immune system through cleavage of the Fc portion of antigen-bound IgG [20], [21], properdin [18], and complement C3 [19], thereby contributing to bacterial evasion of antibody- and complement-mediated opsonophagocytosis.

Previous studies indicate that opsonizing antibodies specific for M and M-like proteins of GAS are capable of providing protection against GAS infections [34]. Through antibody-induced complement activation followed by opsonization by C3b and its cleavage fragment iC3b, complement receptor 3 (CR3; CD11b/CD18) on phagocytes efficiently takes up and eliminates GAS [35]. SPE B cleaved GAS-bound opsonizing antibodies resulting in failure of complement deposition as well as GAS elimination, enhancing bacterial dissemination. In this study, we showed that the C-terminal domain amino acid residues 345–398 was the major binding site of SPE B with immunoglobulins (Figure 4) using an ELISA binding assay. The results of inhibition ELISA indicated that protein A could inhibit IgG binding to rSPE B_345–398_ (Figure 5A and 5B). The results of the Fc fragment-direct binding assay also indicated that rSPE B_345–398_ bound the Fc portion of IgG; even the binding activity of the Fc fragment of IgG to rSPE B_345–398_ was weaker than that to the zymogen form C192S (Figure 5C). The recombinant protein, rSPE B_345–398_, could efficiently block cleavage of immunoglobulins by mSPE B (Figure 6) and interfere with mSPE B-induced inhibition of complement activation (Figure 7). These functional assays indicate that the C-terminal domain of SPE B is the actual binding site for immunoglobulins. This finding is consistent with the study reported by Wang et al., which showed, by nuclear magnetic resonance spectroscopy, that the flexible C-terminal loop of SPE B may play an important role in controlling substrate binding, resulting in its broad substrate specificity [36].

Clinical investigations indicate that GAS not only induces acute invasive infection but also causes immune-mediated post-infectious sequelae, such as acute rheumatic fever and post-streptococcal glomerulonephritis [37], [38]. Molecular mimicry between group A streptococcus and host antigens has an important role in the development of post-streptococcal sequelae, including glomerulonephritis and rheumatic heart disease. Luo et al. indicate that anti-SPE B antibodies exhibit characteristics of autoantibodies, which cross-react with endothelial cells, causing apoptotic cell death in the mouse heart valve. They further identify the SPE B antigenic epitope recognized by anti-SPE B antibodies, indicating that the major epitope of anti-SPE B antibodies (mAb 10G) is localized to amino-acid residues 296–310 of SPE B [39]. Amino-acid residues 296–310, the probable epitope of the autoantigen, were distinct from amino-acid residues 345–398 of SPE B. Even rSPE B_281–358_ seemed to manifest binding activity with IgG (Figure 4A). We excluded the probable epitope of the autoantigen and based on the results of the ELISA binding assay and functional assays, we used the recombinant protein rSPE B_345–398_ to immunize BALB/c mice and found that rSPE B_345–398_-immunized mice could effectively protect mice from skin lesions and GAS NZ131-induced death (Figure 8).

Using rSPE B_345–398_ to immunize mice, we did not find characteristics of glomerulonephritis in immunized mice (Figure S1). The serum creatinine (CRTN) and blood urea nitrogen (BUN) levels of rSPE B_345–398_-immunized group (CRTN: 0.46±0.05 mg/dl; BUN: 20.5±2.38 mg/dl) were similar to the control group (CRTN: 0.44±0.04 mg/dl; BUN: 21.8±2.48 mg/dl). Those results indicate that immunization of rSPE B_345–398_ was able to protect mice from GAS infection but not to induce autoimmune-like symptoms. Furthermore, six synthetic peptides which contain C-terminal region extended from residues 281 to 398 of SPE B were examined by IgG binding assay. ELISA results indicated the C-terminal motifs of SPE B, Gly^346^-Gly^360^ and Ala^376^-Pro^398^, both mediated the binding of IgG. Immunization with synthetic peptides either Gly^346^-Gly^360^ or Ala^376^-Pro^398^ protected mice against challenge with a lethal dose of GAS (unpublished data). In this study, we used the NZ131 GAS strain to infect mice, while the protective effects of rSPE B_345–398_ on other GAS clinical isolates need further investigation.

## Supporting Information

Figure S1 **Histological examination of kidneys from mice immunized with rSPE B_345–398._** BALB/c mice were immunized four times with PBS (A) or C-terminal domain of SPE B; rSPE B_345–398_ (B), and their kidney sections were stained with hematoxylin-eosin (n = 4 per group). Scale bar, 50 µm.
(TIF)
