# Supplementary figures and images for: Determining Antibody-Binding Site of Streptococcal Pyrogenic Exotoxin B to Protect Mice from Group A Streptococcus Infection
Source: PLoS One. 2013 Jan 31;8(1):e55028. doi: 10.1371/journal.pone.0055028 (PMC3561455; doi:10.1371/journal.pone.0055028)

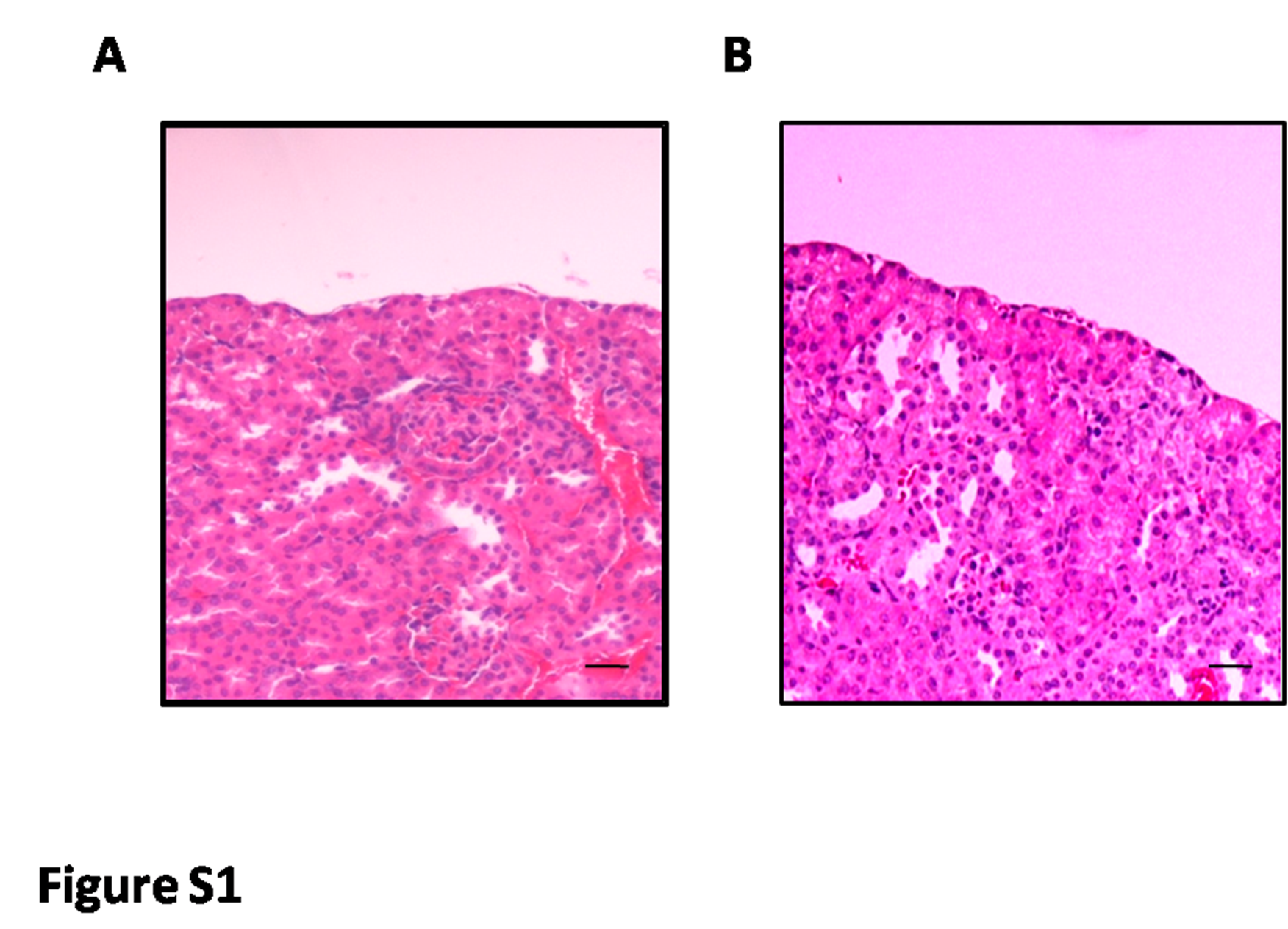

Supplement: Figure S1 — Histological examination of kidneys from mice immunized with rSPE B345–398. BALB/c mice were immunized four times with PBS (A) or C-terminal domain of SPE B; rSPE B345–398 (B), and their kidney sections were stained with hematoxylin-eosin (n = 4 per group). Scale bar, 50 µm. (TIF) [file pone.0055028.s001.tif]
